# Supplementary material for: Specific glycine-dependent enzyme motion determines the potency of conformation selective inhibitors of threonyl-tRNA synthetase
Source: Commun Biol. 2024 Jul 16;7:867. doi: 10.1038/s42003-024-06559-x (PMC11252418; doi:10.1038/s42003-024-06559-x)
Supplement: Supplementary file 3 — Description of Additional Supplementary Materials [file 42003_2024_6559_MOESM3_ESM.pdf]

# Description of Additional Supplementary Files

**File name:** Supplementary Data 1

**Description:** The source data behind the graphs in the paper

**File name:** Supplementary Movie 1

**Description:** This movie shows the different dynamic behaviours of EcThrRS_WT (shown as blue ribbon) and G463S (shown as light cyan ribbon) compared to a static apo state structure (shown as pink ribbon). Residues 422–448 are highlighted in red. During MD simulation, the WT underwent larger conformational changes, while the G463S tended to remain in a closed state and move within a smaller range.

**File name:** Supplementary Movie 2

**Description:** This movie shows the proposed binding process of OB to EcThrRS_WT. EcThrRS_WT undergoes larger conformational changes, which allow OB to bind the tRNA site instead of the ATP site to form a covalent bond with Tyr462 and prevent ThrRS from binding ATP. The EcThrRS_G463A–OB (PDB code: 8WII) and EcThrRS_WT–OB (PDB code: 8H98) complex structures were used as models for morph operation. This movie was prepared with ChimeraX (https://www.cgl.ucsf.edu/chimerax).

**File name:** Supplementary Movie 3

**Description:** This movie shows the proposed unstable binding of OB to EcThrRS_G463A. Because the structure of this mutant is relatively rigid, OB cannot reach the tRNA A76 binding site. When it binds to ThrRS's ATP site, it can be competed out by ATP. The EcThrRS_G463A–OB (PDB code: 8WII) and EcThrRS_G463A–ATP (PDB code: 8WIH) complex structures were used as models for morph operation. This movie was prepared with ChimeraX (https://www.cgl.ucsf.edu/chimerax).
