# Supplementary material for: Specific glycine-dependent enzyme motion determines the potency of conformation selective inhibitors of threonyl-tRNA synthetase
Source: Commun Biol. 2024 Jul 16;7:867. doi: 10.1038/s42003-024-06559-x (PMC11252418; doi:10.1038/s42003-024-06559-x)
Supplement: Supplementary file 2 — Supplementary Information [file 42003_2024_6559_MOESM2_ESM.pdf]

Supplementary Information for

Specific Glycine Dependent Enzyme Motion Determines the Potency of Conformation Selective Inhibitors of Threonyl-tRNA Synthetase

Hang Qiao[1,#], Zilu Wang[1,#], Hao Yang[2,#], Mingyu Xia[1], Guang Yang[3], Fang Bai[2,4,5*], Jing Wang[1,3*], Pengfei Fang[1,3,6*]

CONTENTS

I. Legends for Supplementary Movies......3

II. Supplementary Tables 4

Supplementary Table 1......4

Supplementary Table 2......5

Supplementary Table 3......6

Supplementary Table 4......7

Supplementary Table 5......8

III. Supplementary Figures......9

Supplementary Figure 1 9

Supplementary Figure 2 11

Supplementary Figure 3 13

Supplementary Figure 4 14

Supplementary Figure 5 15

Supplementary Figure 6 16

Supplementary Figure 7 17

Supplementary Figure 8 18

Supplementary Figure 9 19

Supplementary Figure 10 20

Supplementary Figure 11 21

Supplementary Figure 12 22

Supplementary Figure 13 23

Supplementary Figure 14 24

Supplementary Figure 15 25

Supplementary Figure 16 26

Supplementary Figure 17 27

IV. Supplementary Note 1 28

Key resources table 28

Coding sequence of *Ec*ThrRS_WT 31

Protein sequence of *Ec*ThrRS_WT 31

Coding sequence of ObaO_WT 32

Protein sequence of ObaO_WT 32

Abbreviations 33

V. Supplementary References..34

I. Legends for Supplementary Movies

Supplementary Movie 1

This movie shows the different dynamic behaviours of *Ec*ThrRS_WT (shown as blue ribbon) and G463S (shown as light cyan ribbon) compared to a static *apo* state structure (shown as pink ribbon). Residues 422–448 are highlighted in red. During MD simulation, the WT underwent larger conformational changes, while the G463S tended to remain in a closed state and move within a smaller range.

Supplementary Movie 2

This movie shows the proposed binding process of OB to *Ec*ThrRS_WT. *Ec*ThrRS_WT undergoes larger conformational changes, which allow OB to bind the tRNA site instead of the ATP site to form a covalent bond with Tyr462 and prevent ThrRS from binding ATP. The *Ec*ThrRS_G463A–OB (PDB code: 8WII) and *Ec*ThrRS_WT–OB (PDB code: 8H98) complex structures were used as models for morph operation. This movie was prepared with ChimeraX (https://www.cgl.ucsf.edu/chimerax).

Supplementary Movie 3

This movie shows the proposed unstable binding of OB to EcThrRS_G463A. Because the structure of this mutant is relatively rigid, OB cannot reach the tRNA A76 binding site. When it binds to ThrRS's ATP site, it can be competed out by ATP. The *Ec*ThrRS_G463A–OB (PDB code: 8WII) and EcThrRS_G463A–ATP (PDB code: 8WIH) complex structures were used as models for morph operation. This movie was prepared with ChimeraX (https://www.cgl.ucsf.edu/chimerax).

II. Supplementary Tables

Supplementary Table 1. Statistics of X-ray crystallographic data collection and model refinements of *Ec*ThrRS_G463S.

	*Ec*ThrRS_G463S
**PDB code**	8WIA
**Data collection**	
Space group	$P2_12_12_1$
Cell dimensions	
a, *b*, *c* (Å)	90.96, 107.41, 112.80
α, β, γ (°)	90.00, 90.00, 90.00
Resolution (Å)	42.18-1.96 (2.03-1.96)*
R_{sym} or R_{merge} (%)	8.9 (92.2)
I / s*I*	12.9 (2.5)
Completeness (%)	97.2 (99.9)
Redundancy	7.3 (7.5)
**Refinement**	
No. reflections	77735 (7919)
R_{work} / R_{free} (%)	21.3/23.8
No. atoms	
Protein	6551
Metal	2
Solvent	574
B-factors (Å^2)	
Protein	37.05
Metal	37.68
Solvent	41.93
R.m.s. deviations	
Bond lengths (Å)	0.002
Bond angles (°)	0.48
Ramachandran plot	
Most favored [%]	99.62
Additional allowed [%]	0.38

*Values in parentheses are for the highest-resolution shell.

Supplementary Table 2. Statistics of X-ray crystallographic data collection and model refinements of *Ec*ThrRS_L489M–OB.

	*Ec*ThrRS_L489M–OB
**PDB code**	8WIJ
**Data collection**	
Space group	$P2_12_12_1$
Cell dimensions	
a, *b*, *c* (Å)	103.25, 84.24, 102.59
α, β, γ (°)	90.00, 90.00, 90.00
Resolution (Å)	36.39-3.08 (3.19-3.08)*
R_{sym} or R_{merge} (%)	19.0 (105.1)
I / s*I*	9.4 (2.6)
Completeness (%)	91.3 (99.7)
Redundancy	6.4 (6.7)
**Refinement**	
No. reflections	15640 (1680)
R_{work} / R_{free} (%)	24.0/28.0
No. atoms	
Protein	6515
Ligand	52
Metal	2
Solvent	12
B-factors (Å^2)	
Protein	70.12
Ligand	76.72
Metal	61.70
Solvent	60.87
R.m.s. deviations	
Bond lengths (Å)	0.002
Bond angles (°)	0.45
Ramachandran plot	
Most favored [%]	98.74
Additional allowed [%]	1.26

*Values in parentheses are for the highest-resolution shell.

Supplementary Table 3. Statistics of X-ray crystallographic data collection and model refinements of *Ec*ThrRS_G463S_Q484A.

	*Ec*ThrRS_G463S_Q484A
**PDB code**	8WIG
**Data collection**	
Space group	$P2_12_12_1$
Cell dimensions	
a, *b*, *c* (Å)	91.14, 108.11, 113.50
α, β, γ (°)	90.00, 90.00, 90.00
Resolution (Å)	56.21-3.22 (3.33-3.22)*
R_{sym} or R_{merge} (%)	11.4 (63.9)
I / s*I*	10.6 (2.5)
Completeness (%)	99.6 (99.8)
Redundancy	4.6 (4.5)
**Refinement**	
No. reflections	18371 (1808)
R_{work} / R_{free} (%)	21.0/22.4
No. atoms	
Protein	6539
Metal	2
B-factors (Å^2)	
Protein	68.44
Metal	60.14
R.m.s. deviations	
Bond lengths (Å)	0.002
Bond angles (°)	0.43
Ramachandran plot	
Most favored [%]	99.25
Additional allowed [%]	0.75

*Values in parentheses are for the highest-resolution shell.

Supplementary Table 4. Statistics of X-ray crystallographic data collection and model refinements of *Ec*ThrRS_G463A–OB.

	*Ec*ThrRS_G463A–OB
**PDB code**	8WII
**Data collection**	
Space group	$P2_12_12_1$
Cell dimensions	
a, *b*, *c* (Å)	90.40, 108.81, 113.77
α, β, γ (°)	90.00, 90.00, 90.00
Resolution (Å)	78.64-2.98 (3.08-2.98)*
R_{sym} or R_{merge} (%)	13.1 (80.3)
I / s*I*	6.8 (2.4)
Completeness (%)	99.3 (99.5)
Redundancy	4.2 (4.4)
**Refinement**	
No. reflections	23476 (2313)
R_{work} / R_{free} (%)	0.250 (0.301)
No. atoms	
Protein	6545
Ligand	52
Metal	2
B-factors (Å^2)	
Protein	69.00
Ligand	92.53
Metal	81.58
R.m.s. deviations	
Bond lengths (Å)	0.007
Bond angles (°)	1.24
Ramachandran plot	
Most favored [%]	98.25
Additional allowed [%]	1.75

*Values in parentheses are for the highest-resolution shell.

Supplementary Table 5. Statistics of X-ray crystallographic data collection and model refinements of *Ec*ThrRS_G463A-ATP.

	*Ec*ThrRS_G463A–ATP
**PDB code**	8WIH
**Data collection**	
Space group	$P2_12_12_1$
Cell dimensions	
a, *b*, *c* (Å)	87.18, 109.38, 113.79
α, β, γ (°)	90.00, 90.00, 90.00
Resolution (Å)	50.48-2.44 (2.52-2.44)*
R_{sym} or R_{merge} (%)	11.3 (81.4)
I / s*I*	14.2 (2.8)
Completeness (%)	88.6 (96.5)
Redundancy	8.9 (6.6)
**Refinement**	
No. reflections	36683 (3927)
R_{work} / R_{free} (%)	24.2 (26.4)
No. atoms	
Protein	6398
Ligand	50
Metal	2
Solvent	48
B-factors (Å^2)	
Protein	46.77
Ligand	40.30
Metal	38.45
Solvent	43.72
R.m.s. deviations	
Bond lengths (Å)	0.003
Bond angles (°)	0.56
Ramachandran plot	
Most favored [%]	98.87
Additional allowed [%]	1.13

*Values in parentheses are for the highest-resolution shell.

III. Supplementary Figures

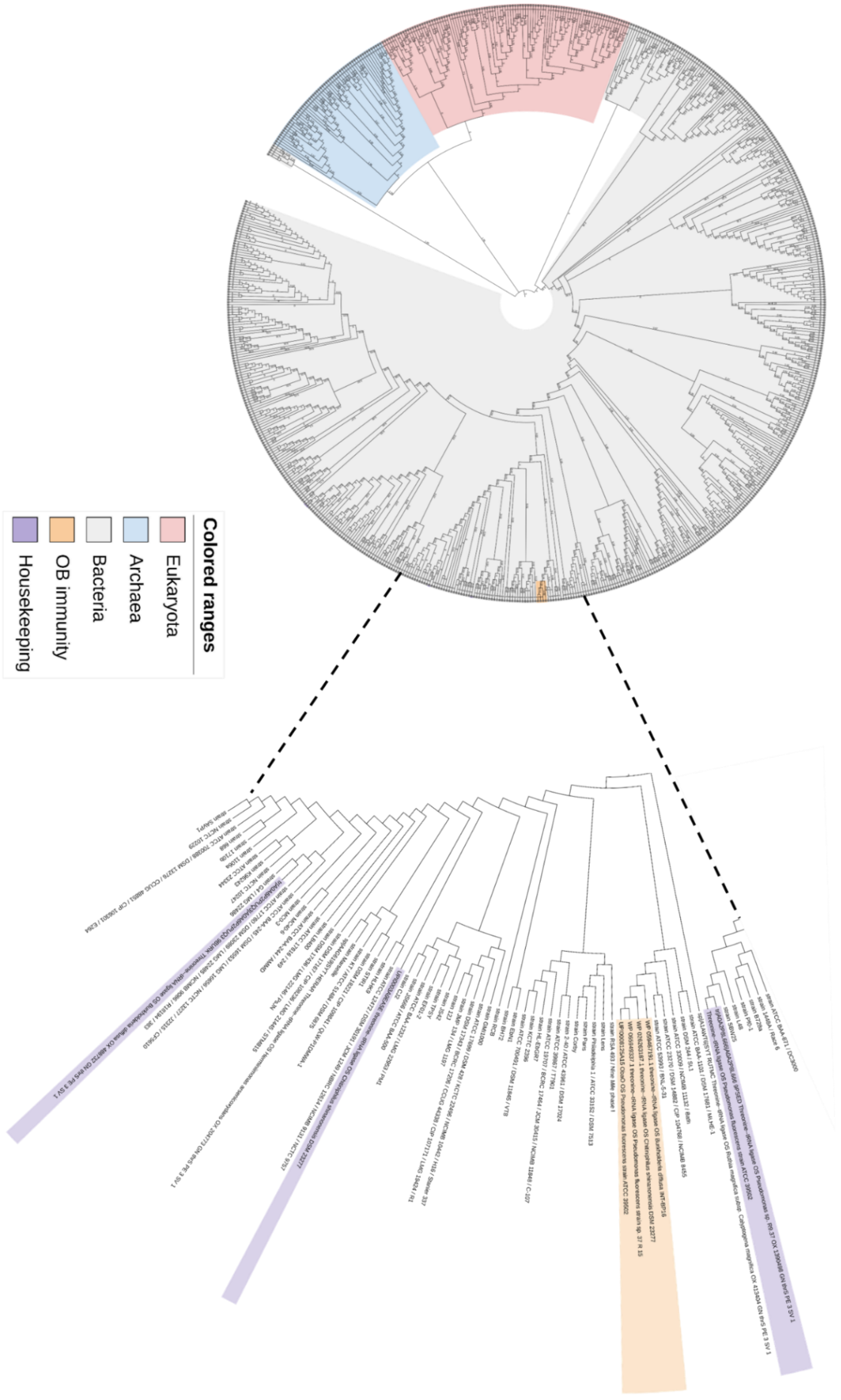

Supplementary Figure 1. OB-resistant ThrRSs are more closely related to each other than to their housekeeping ThrRS equivalents.

Maximum likelihood tree of ThrRS amino acid sequences. 4 putative OB immune proteins clade with each other rather than housekeeping ThrRSs from the same species. The tree was constructed using Swiss-Prot reviewed ThrRS amino acid sequences retrieved from UniProtKB[1] using the ENZYME[2] entry identifier E.C.6.1.1.3, along with the nonredundant amino acid sequences of ObaO (*Pseudomonas fluorescens* ATCC 39502, NCBI entry: KX931446.1) and another 3 homologs identified from OB BGCs (*Pseudomonas fluorescens* ATCC 37 R 15, NCBI entry: NZ_CVTV01000010.1; *Burkholderia diffusa* INT-BP16, NCBI entry: NZ_LOUS01000041.1; *Chitiniphilus shinanonensis* DSM 23277, PDB entry: NZ_KB895358.1). MEGA7[3] was applied to generate a variant phylogenetic tree and the tree was represented using iTOL v6[4].

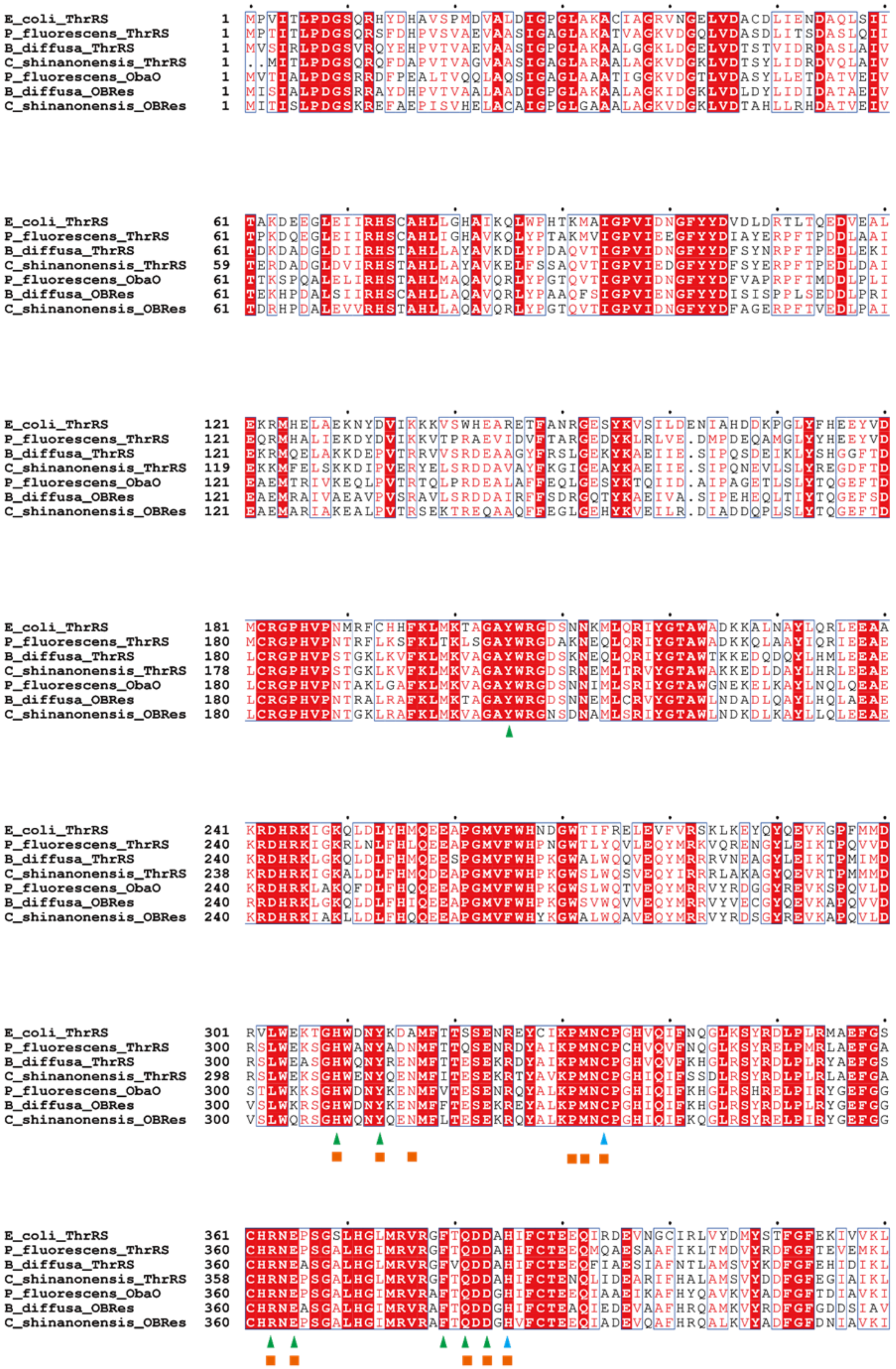
E_coli_ThrRS
P_fluorescens_ThrRS
B_diffusa_ThrRS
C_shinanonensis_ThrRS
P_fluorescens_ObaO
B_diffusa_OBRes
C_shinanonensis_OBRes

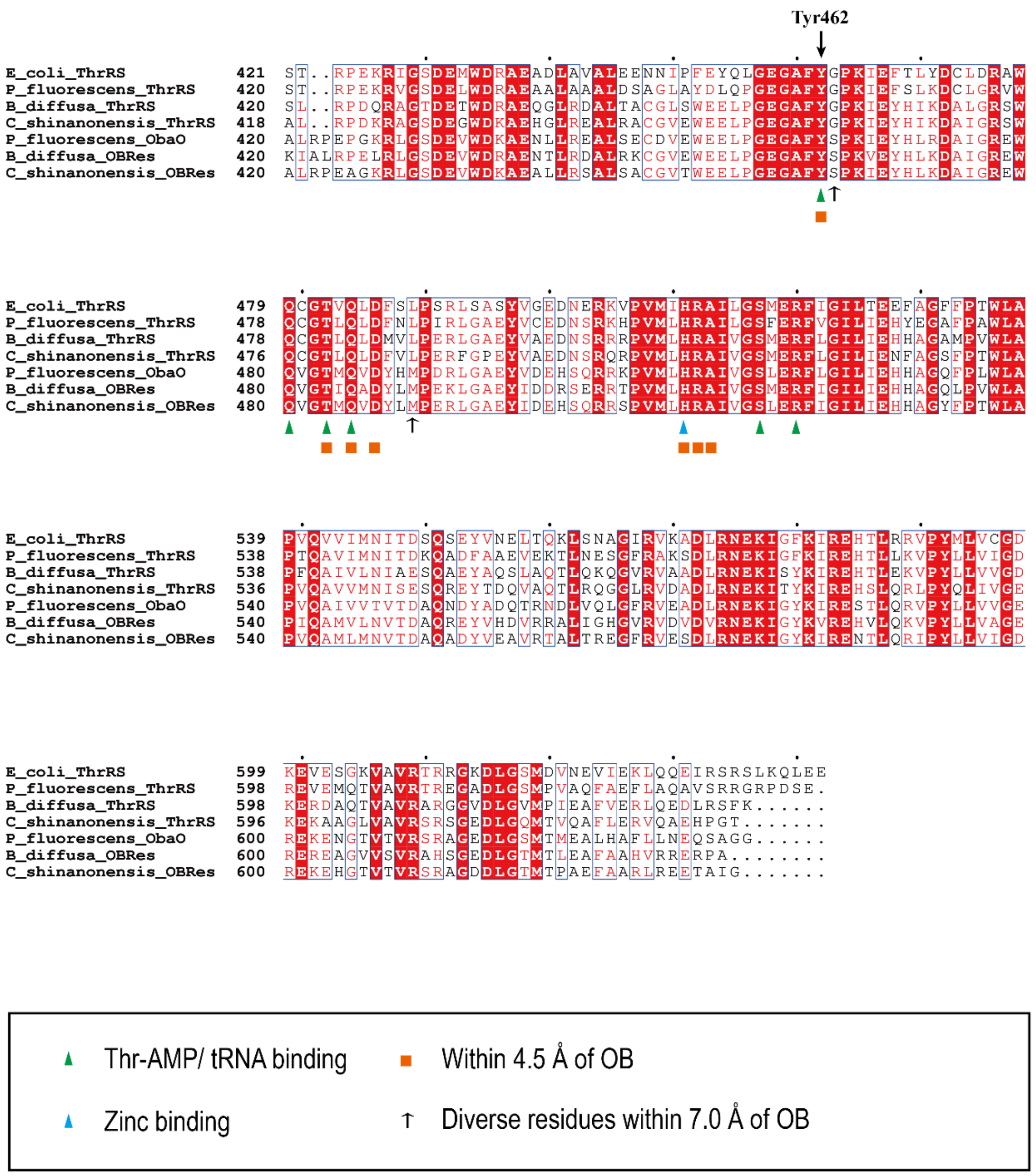

Supplementary Figure 2. Multiple sequence alignment of ThrRS homologs.

The Sequence alignment was generated using *Clustal Omega*[5] and displayed by *Espript 3.0*[6]. ThrRS residues that interact with Thr-AMP and tRNA substrates or the catalytic Zn^{2+} ion are indicated with green triangles and blue triangles, respectively. Residues within 4.5 Å of OB are indicated with orange squares. Nonconserved residues of interest within 7.0 Å of OB are indicated with black arrows.

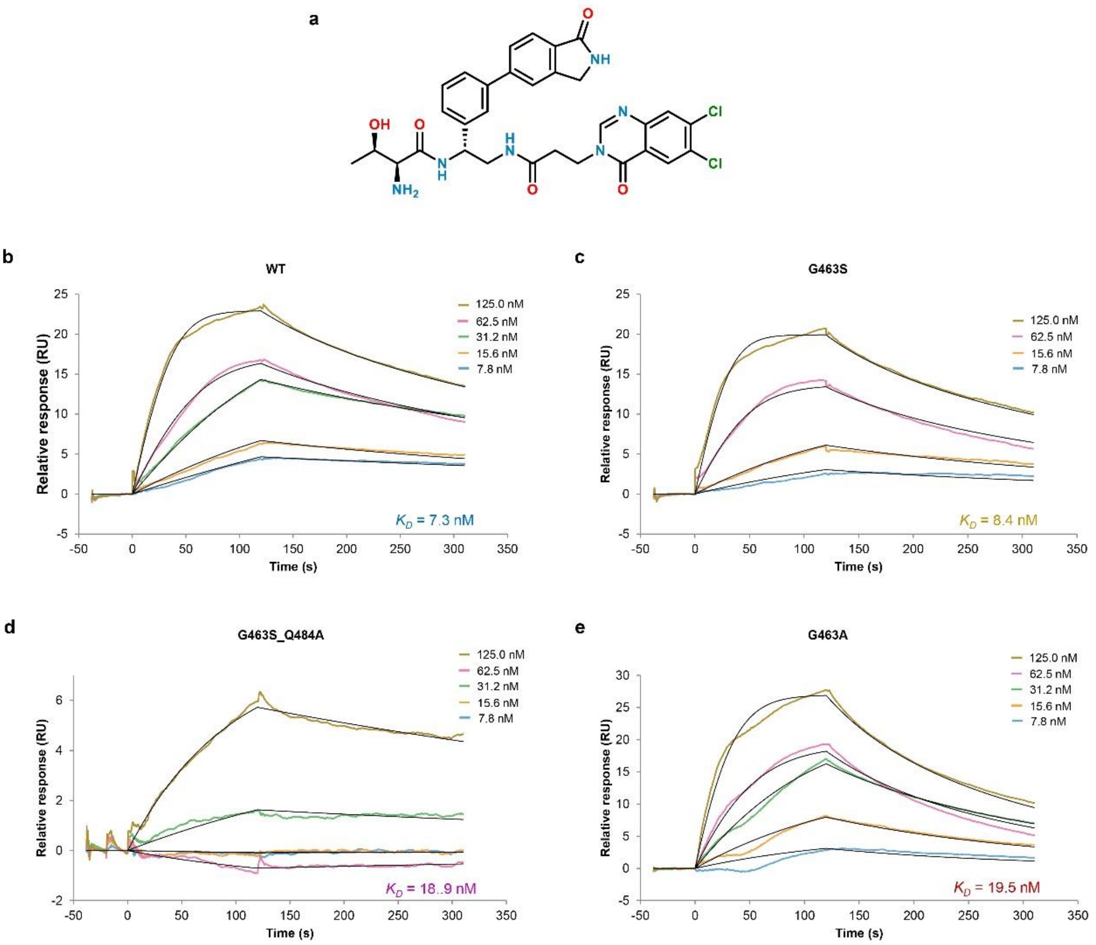

Supplementary Figure 3. 36j retains strong binding affinity for *Ec*ThrRS_WT, G463S, G463S_Q484A and G463A.

(a) Chemical structures of compound **36j**. **(b-e)** Surface plasmon resonance-based binding curves for **36j** binding to *Ec*ThrRS_WT, G463S, G463_Q484A and G463A. **36j** was set in a twofold dilution series from 125 nM to 7.8 nM (n = 1 for each series). Black lines show the fitting to a one-to-one kinetic binding model, RU, response unit. **(b)** *Ec*ThrRS_WT. Association rate constant (*ka*) = $5.56 \times 10^5 \cdot M^{-1} \cdot s^{-1}$ and dissociation rate constant (*kd*) = $4.08 \times 10^{-3} \cdot s^{-1}$, K_D = 7.34×10^{-9} M, Rmax = 32.4 Ru, $\chi^2 = 9.4 \times 10^{-2}$ RU^2. **(c)** *Ec*ThrRS_G463S. *ka* = $6.87 \times 10^5 \cdot M^{-1}$ s^{-1} and kd = $5.78 \times 10^{-3} \cdot s^{-1}$, K_D = 8.41×10^{-9} M, Rmax = 45.3 Ru, $\chi^2 = 1.9 \times 10^{-1}$ RU^2. **(d)** *Ec*ThrRS_G463S_Q484A. *ka* = $7.39 \times 10^4 \cdot M^{-1} \cdot s^{-1}$ and *kd* = $1.44 \times 10^{-3} \cdot s^{-1}$, K_D = 1.95×10^{-8} M, Rmax = 15.0 Ru, $\chi^2 = 2.0 \times 10^{-2}$ RU^2. **(e)** *Ec*ThrRS_G463A. *ka* = $4.46 \times 10^5 \cdot M^{-1} \cdot s^{-1}$ and *kd* = $8.45 \times 10^{-3} \cdot s^{-1}$, K_D = 1.89×10^{-8} M, Rmax = 39.5 Ru, $\chi^2 = 4.4 \times 10^{-1}$ RU^2. These data confirmed that **36j** retains strong binding affinity for the key mutants in this study.

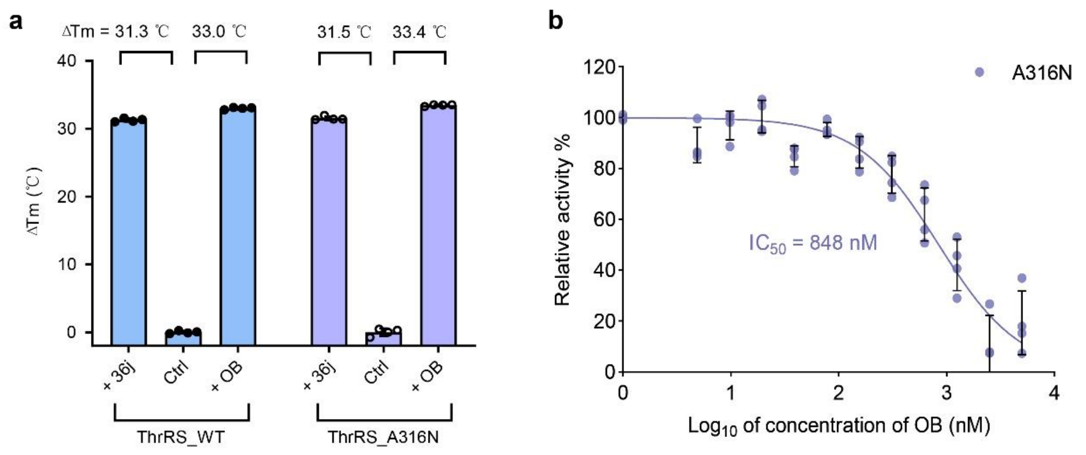

Supplementary Figure 4. Mutation of Ala316 to asparagine does not confer resistance of OB to *Ec*ThrRS.

(a) Diagram of the ΔTm values of *Ec*ThrRS_WT and *Ec*ThrRS_A316N in the presence or absence of OB and 36j. Evaluations were carried out in four repeats, and error bars indicate the respective standard deviation (n = 4, mean value ± SD). All the data points are shown as small circles. **(b)** The inhibitory curve of OB on the ATP hydrolysis activity of *Ec*ThrRS_A316N. Evaluations were carried out in four repeats, and error bars indicate the respective standard deviation (n = 4, mean value ± SD). All the data points for *Ec*ThrRS_A316N are shown as pale purple dots.

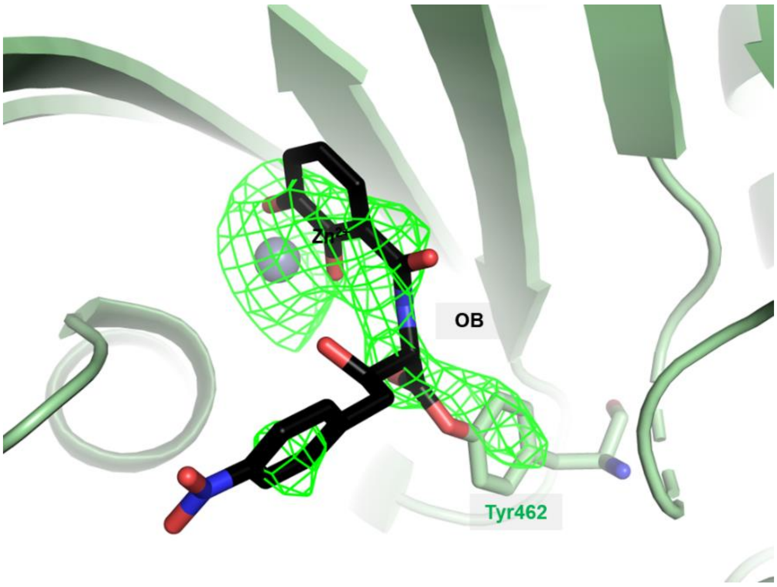

Supplementary Figure 5. *Ec*ThrRS_L489M can be covalently inhibited by OB.
The Fo-Fc electron density was calculated with the OB-omitted structure model (made from the *Ec*ThrRS_L489M OB structure), contoured at 3.0 σ, and is shown as green meshes.

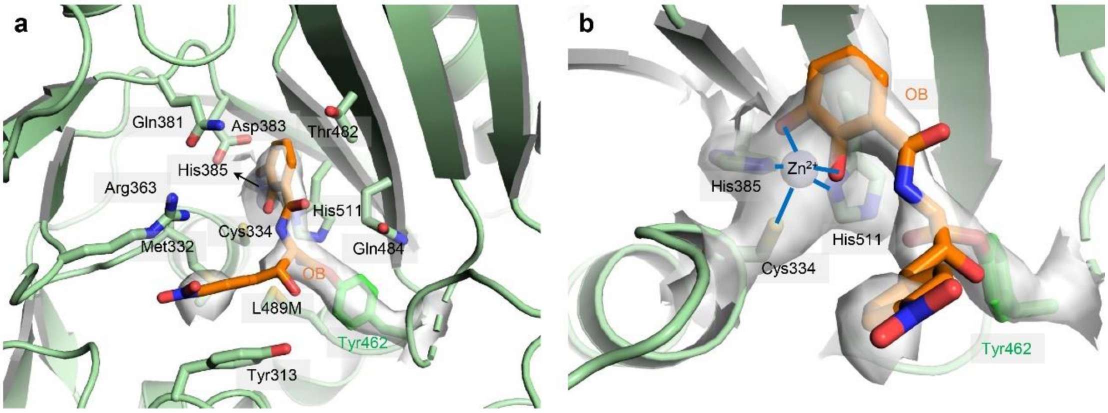

Supplementary Figure 6. The binding mode of OB in the catalytic pocket of *Ec*ThrRS_L489M.

(a) The catalytic center of *Ec*ThrRS_L489M with bound OB. The phenolic group of Tyr462 (green sticks) forms a new ester bond with OB (orange sticks). Interacting residues are shown as sticks. The 2Fo-Fc electron density of Tyr462–OB (contoured at 1.0 σ) is shown as gray transparent surface. **(b)** Close-up view of residues coordinated with a Zn^{2+} ion. Two hydroxyl groups on the *o*-diphenol moiety of OB coordinate with a Zn^{2+} ion. Residues Cys334, His385, His511, Tyr462 and OB are shown as sticks. The 2Fo-Fc electron density of these residues (contoured at 1.0 σ) is shown as a transparent surface.

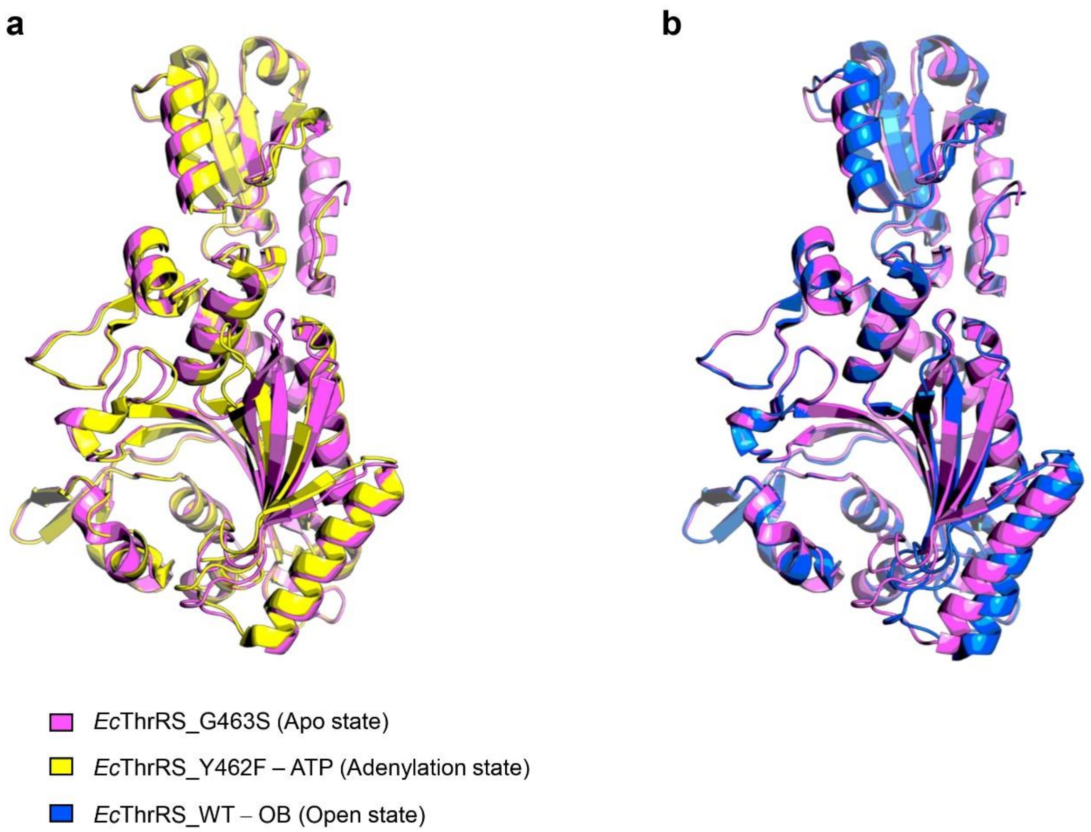

Supplementary Figure 7. The catalytic pocket of *Ec*ThrRS slightly tightens when it binds to ATP and extends when it binds to OB.

(a) Superimposition of the structures of *Ec*ThrRS_Y462F–ATP (PDB code: 8H99, yellow cartoons) and *Ec*ThrRS_G463S (violet cartoons). **(b)** Superimposition of the structures of *Ec*ThrRS_WT–OB (PDB code: 8H98, marine cartoons) and *Ec*ThrRS_G463S (violet cartoons).

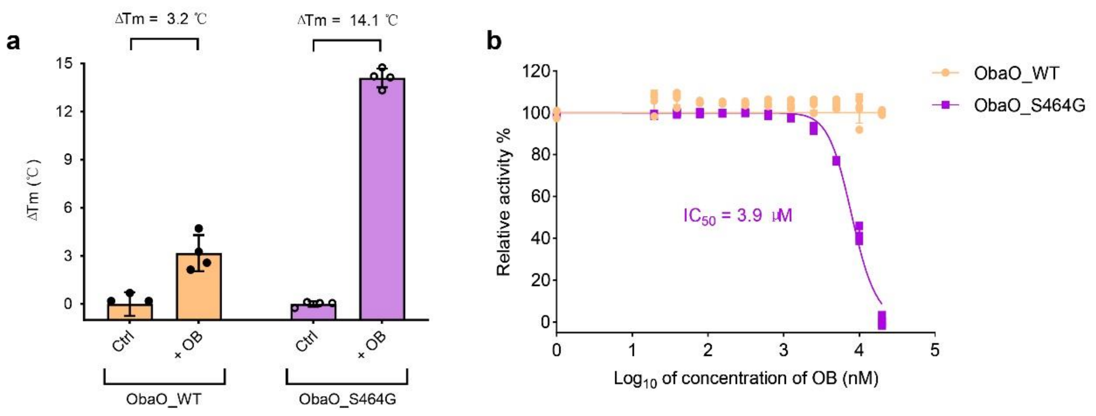

Supplementary Figure 8. Mutation of Ser464 of ObaO to glycine results in sensitivity to OB.

(a) Diagram of the ΔTm values of ObaO_WT and ObaO_S464G in the presence or absence of OB and 36j. Evaluations were carried out in four repeats, and error bars indicate the respective standard deviation (n = 4, mean value ± SD). All the data points are shown as small circles. **(b)** Inhibitory curves of OB on the ATP hydrolysis activity of ObaO_WT and ObaO_S464G. Evaluations were carried out in four repeats, and error bars indicate the respective standard deviation (n = 4, mean value ± SD). All the data points for ObaO_WT and ObaO_S464G are shown as violet dots and orange dots, respectively.

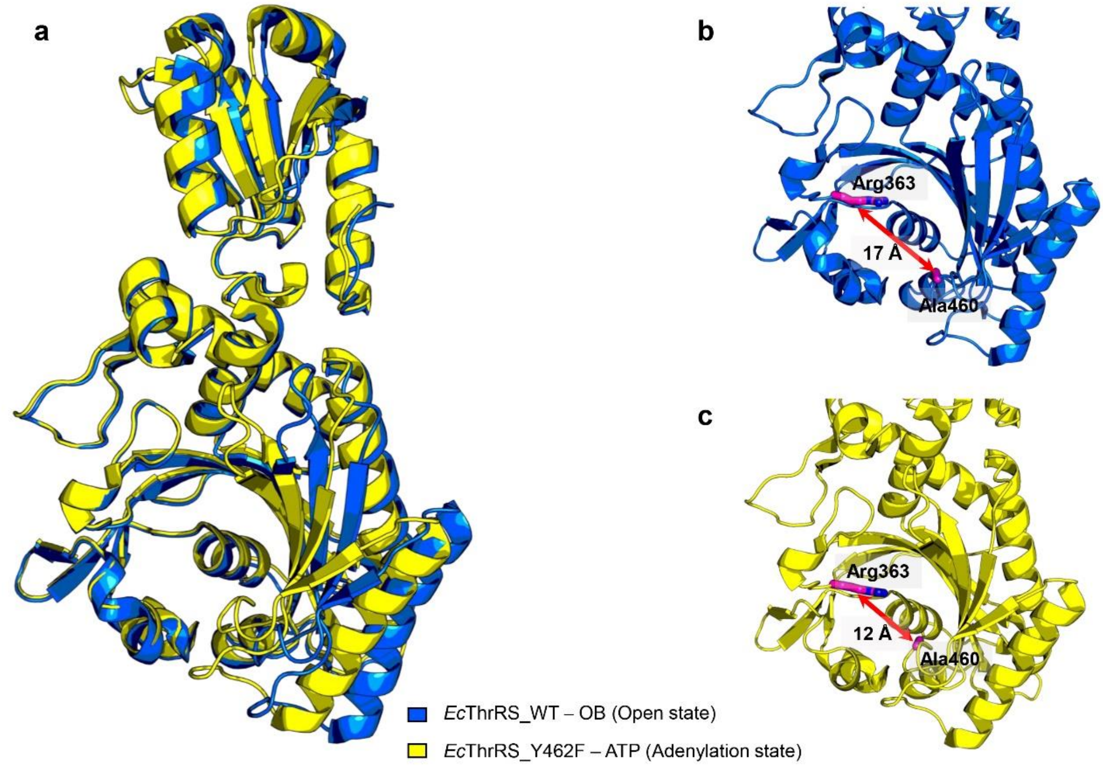

Supplementary Figure 9. The distance between the centroids of Arg363 and Ala460 of *Ec*ThrRS changes when the protein binds different ligands.

(a) Superimposition of the structures of *Ec*ThrRS_WT–OB (PDB:8H98, marine cartoons) and *Ec*ThrRS_Y462F–ATP (PDB: 8H99, yellow cartoons). **(b)** Zoomed-in view of the catalytic pocket of *Ec*ThrRS_WT–OB. Arg363 and Ala460 are shown as violet sticks. The distances between the centroids of the two residues are shown as red directional arrows. **(c)** Zoomed-in view of the catalytic pocket of *Ec*ThrRS_Y462F–ATP. Arg363 and Ala460 are shown as violet sticks. The distances between the centroids of the two residues are shown as red directional arrows.

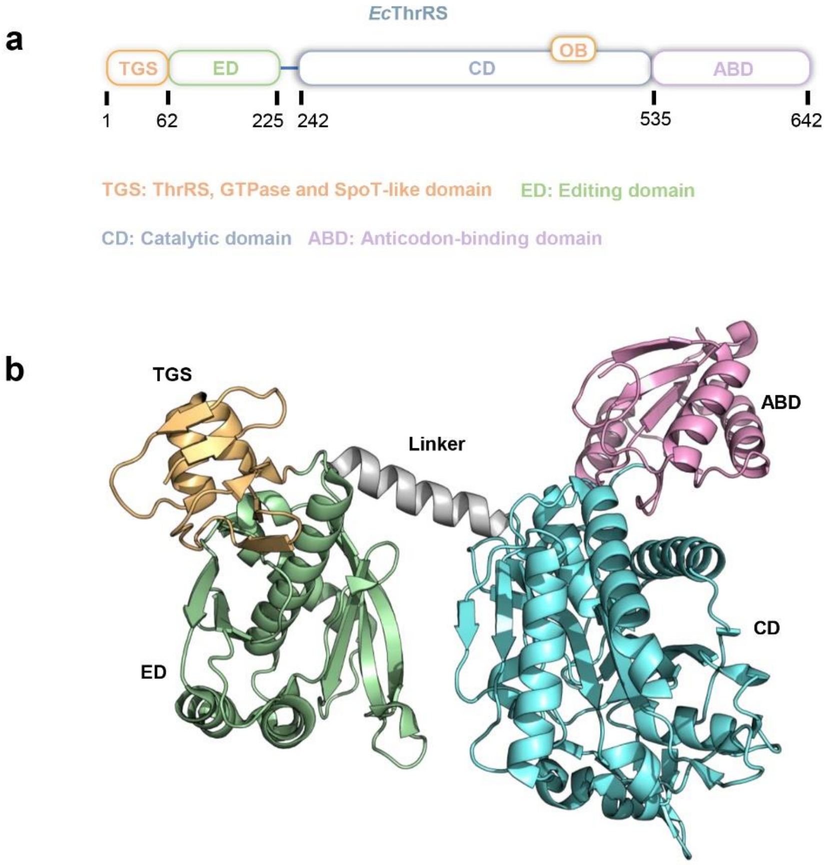

Supplementary Figure 10. Schematic representation of the domain organization of *E. coli* ThrRS.

(a) Domain organization of *Ec*ThrRS. TGS: ThrRS, GTPase and SpoT-like domain; ED: editing domain; CD: catalytic domain; ABD: anticodon-binding domain. **(b)** Structural illustration of *E. coli* ThrRS (PDB code: 1QF6). TGS is shown as wheat cartoons, ED is shown as green cartoons, CD is shown as cyan cartoons, and ABD is shown as pink cartoons. There is an α-helix linker between the ED and CD domains, which divides ThrRS into two relatively independent fragments.

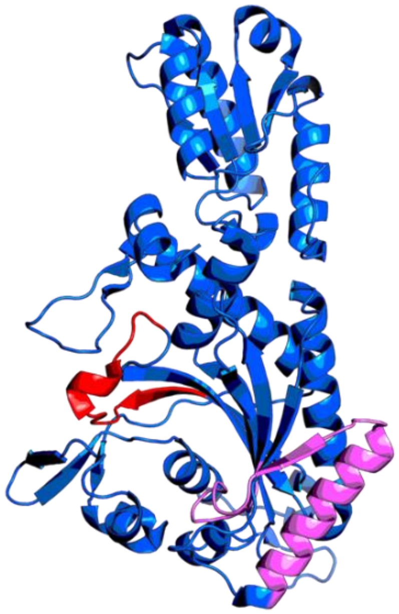

Supplementary Figure 11. Locations of residues 358-373 and residues 432-465 in the *Ec*ThrRS structure.

Residues 358-373 are shown as red cartoons and residues 432-465 are shown as violet cartoons.

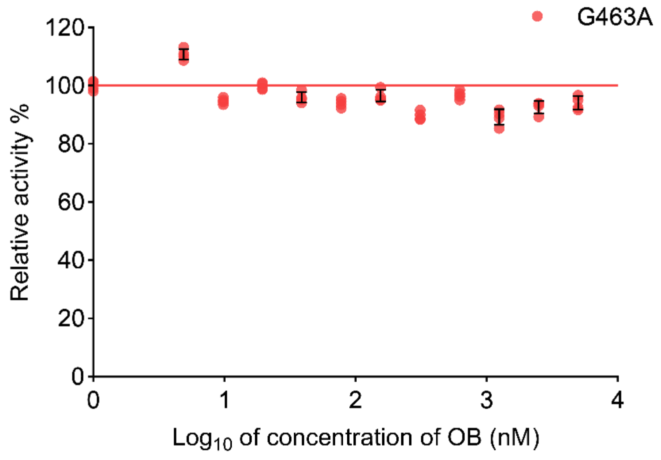

Supplementary Figure 12. The inhibitory curve of OB on the ATP hydrolysis activity of *Ec*ThrRS_G463A.

Evaluations were carried out in four repeats, and error bars indicate the respective standard deviation (n = 4, mean value ± SD). All the data points for *Ec*ThrRS_G463A are shown as red dots.

a **b**

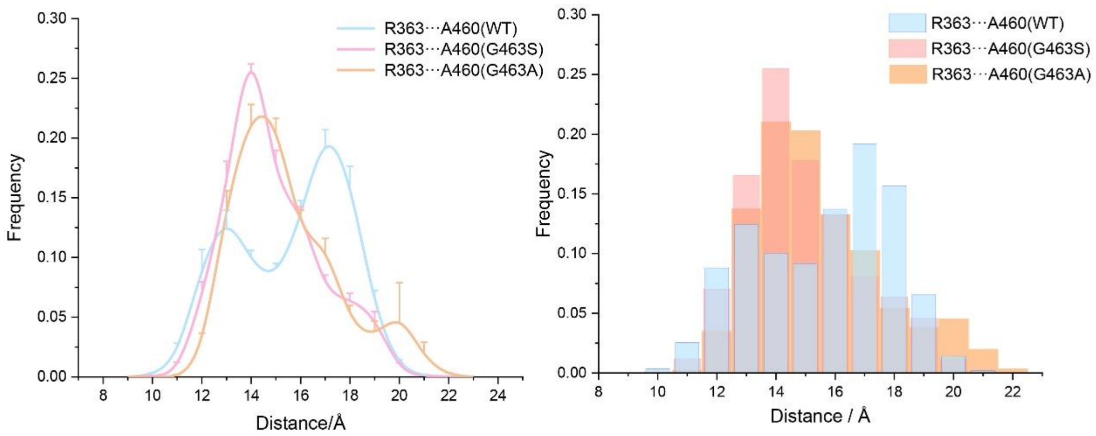

Supplementary Figure 13. Comparison of the conformational spaces of *Ec*ThrRS_WT, G463S and G463A.

The distance between the center masses of the amino acid residues Arg363 and Ala460 is defined as the width of the catalytic pocket. 8 sets of simulations with different random initial velocities were performed, and data were collected from 9 to 23 Å per angstrom. Conformational space shifts (n = 8, mean value ± SD) are shown as curves **(a)** or columns **(b)**.

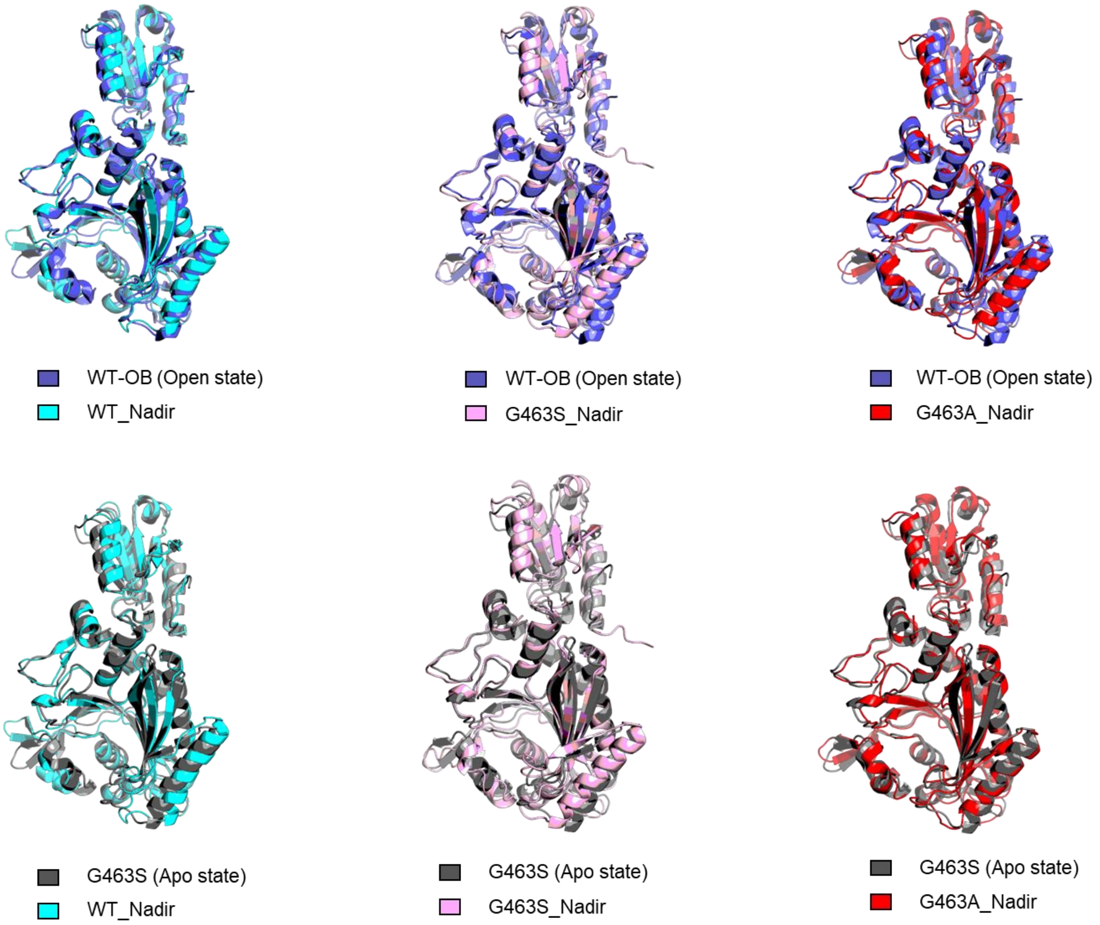

Supplementary Figure 14. **Superimposition of crystal structures with structures taken from the free energy landscape nadir**

Suffix **"**Nadir" indicates the structures taken from the free energy landscape nadir.

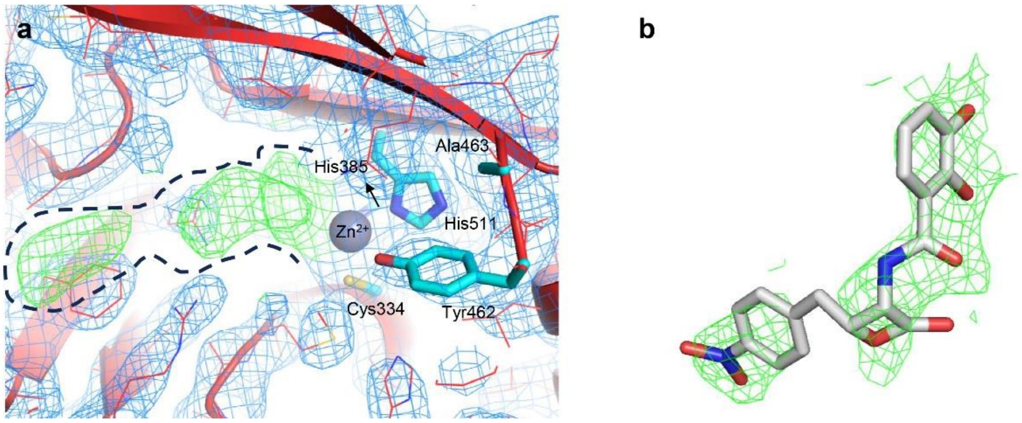

Supplementary Figure 15. An unstable non covalent binding state of OB in *Ec*ThrRS_G463A.

(a) Zoomed-in view of the catalytic pocket of *Ec*ThrRS_G463A, which was crystallized in the presence of OB. The 2Fo-Fc electron map (blue meshes, contoured at 1.0 σ) is shown together with the protein structure model. The Fo-Fc electron map (green meshes, contoured at 3.0 σ) showed positive peaks at the ATP binding site, which is circled by black dashed lines. The Cys334, His385, His511, Tyr462 and Ala463 residues are shown as cyan sticks. **(b)** The unoccupied electron map is filled with the OB structure.

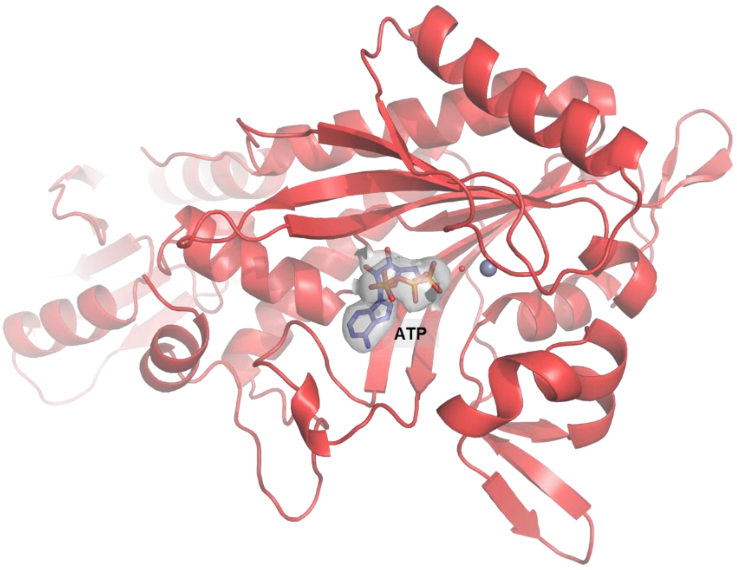

Supplementary Figure 16. *Ec*ThrRS_G463A only binds ATP in the presence of OB and ATP.

The catalytic pocket of *Ec*ThrRS_G463A is shown as red cartoons. ATP is shown as sticks. The 2Fo-Fc electron density of ATP (contoured at 1.0 σ) is shown as a transparent surface.

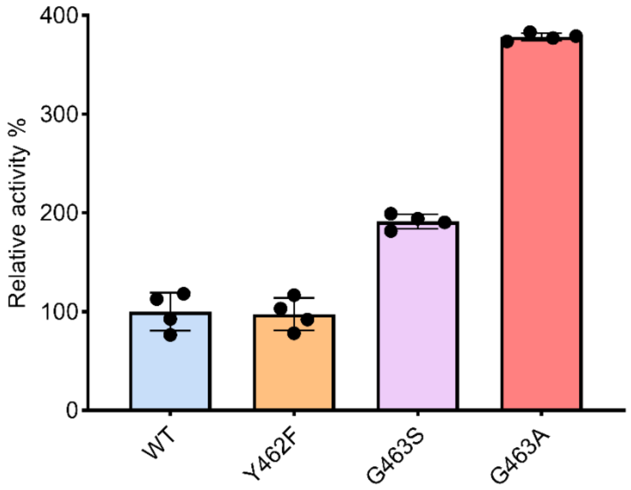

Supplementary Figure 17. Relative ATP hydrolysis rates of *Ec*ThrRS_WT, Y462F, G463S and G463A.

Evaluations were carried out in four repeats, and error bars indicate the respective standard deviation (n = 4, mean value ± SD). The ATP hydrolysis rate of *Ec*ThrRS_WT was normalized to 100%.

IV. Supplementary Note 1

Key resources table

REAGENT	SOURCE	IDENTIFIER
**Bacterial strains**		
BL21 (DE3)	Weidi	CAT#EC1002
DH5α (DE3)	Weidi	CAT#DL1001
**Chemicals, peptides and recombinant proteins**		
obafluorin	GlpBio	GC45617
borrelidin	GlpBio	GC11040
L-threonine	Sigma-Aldrich	T8375
ATP	Sigma-Aldrich	A26209
SYPRO® Orange Dye	Sigma-Aldrich	S5692
**Critical commercial assays**		
Kinase-Glo® Luminescent Kinase Assays	Promega	V6713
Morpheus® I	Molecular Dimension	MD 1-46
**Oligonucleotides**		
**Primers**	**Sequence (5' to 3')**	**Purpose**
*Ec*ThrRS-WT-B	GTGGTGGTGCTCGAGTTCCTCCAATTGTTTAAGACTGCG	For *Ec*ThrRS_WT expression
pET28a-F	CTTAAACAATTGGAGGAACTCGAGCACCACCACCAC	For pET28a vector recombination
pET28a-B	GATTTTACGGTGGTCGCGCATGGTATATCTCCTTCTTAAAGTTAAAC	For pET28a vector recombination
*Ec*ThrRS_G463S-F	AACTGGGTGAAGGCGCTTTCTACTCTCCGAAAA	For *Ec*ThrRS^{G463S} construction
*Ec*ThrRS_G463	TCAATTTTCGGAGAGTAGAAAGCGCCTTC	For EcThrRS^{G463S}

S-B	ACCC	construction
*Ec*ThrRS_L489 M-F	TACAGTACAGCTGGACTTCTCTATGCCGT CTC	For EcThrRS^{L489M} construction
*Ec*ThrRS_L489 M-B	GACGAGACGGCATAGAGAAGTCCAGCTG TACT	For EcThrRS^{L489M} construction
*Ec*ThrRS_G463 S_Q484A-F	GTGCGGTACAGTAGCGCTGGACTTCTCT TTGCC	For EcThrRS^{G463S_Q484A} construction
*Ec*ThrRS_G463 S_Q484A-B	CAAAGAGAAGTCCAGCGCTACTGTACCG CACTGCCA	For EcThrRS^{G463S_Q484A} construction
*Ec*ThrRS_G463 A-F	AGGCGCTTTCTACGCACCGAAAATTGAAT TTACCCT	For EcThrRS^{G463A} construction
*Ec*ThrRS_G463 A-B	AATTCAATTTTCGGTGCGTAGAAAGCGCC TTCACC	For EcThrRS^{G463A} construction
*Ec*ThrRS_A316 N-F	AACTACAAAGATAACATGTTCACCACATCT TCTGA	For EcThrRS^{A316N} construction
*Ec*ThrRS_A316 N-B	GAAGATGTGGTGAACATGTTATCTTTGTA GTTGTC	For EcThrRS^{A316N} construction
ObaO_S464G-F	GAAGGTGCATTCTACGGTCCGAAGATCG AATACCACC	For ObaO^{S464G} construction
ObaO_S464G-B	GTATTCGATCTTCGGACCGTAGAATGCAC CTTCGCCT	For ObaO^{S464G} construction
**Plasmids**		
pET-28a(+)	Protein expression vector used in *E. coli*, encoding N-terminal 6 × His-tag, kanamycin resistance	Novagen
pTRS	pET-28a (+) derivative, containing EcThrRS_242-642_WT_6×His	This study
pTRS-G463S	pET-28a (+) derivative, containing EcThrRS_242-642_ G463S _6×His	This study

pTRS-L489M	pET-28a (+) derivative, containing EcThrRS_242-642_ L489M _6×His	This study
pTRS-G463S_Q484A	pET-28a (+) derivative, containing EcThrRS_242-642_ G463S_Q484A _6×His	This study
pTRS-G463A	pET-28a (+) derivative, containing EcThrRS_242-642_ G463A _6×His	This study
pTRS-A316N	pET-28a (+) derivative, containing EcThrRS_242-642_ A316N _6×His	This study
pObaO-WT	pET-28a (+) derivative, containing ObaO_241-637_6×His	This study
pObaO_S464G	pET-28a (+) derivative, containing ObaO_241-637_S464G_6×His	This study
**Software**		
Jalview	University of Dundee	https://www.jalview.org
XDS	MPI for Medical Research	https://xds.mr.mpg.de
CCP4	Research Complex at Harwell (RCaH), STFC Rutherford Appleton Laboratory, Harwell Science and Innovation Campus	https://www.ccp4.ac.uk
COOT	MRC Laboratory of Molecular Biology	https://www2.mrc-lmb.cam.ac.uk/personal/pemsley/coot
Phenix	University of Cambridge, Duke University, LANL, LBNL	https://phenix-online.org
GraphPad Prism	GraphPad Software Inc	www.graphpad.com
PyMOL	Schrödinger, LLC	www.pymol.org
ChimeraX	University of California San Francisco	https://www.cgl.ucsf.edu/chimerax
Schrödinger	Schrödinger, LLC	https://newsite.schrodinger.com

The sequence of the open reading frame of the pTRS plasmid encoding *Ec*ThrRS_242-642_WT-6×His:

ATGCGCGACCACCGTAAAATCGGTAAACAGCTCGACCTGTACCATATGCAGGAAGAAGCG
CCGGGTATGGTATTCTGGCACAACGACGGCTGGACCATCTTCCGTGAACTGGAAGTGTTT
GTTCGTTCTAAACTGAAAGAGTACCAGTATCAGGAAGTTAAAGGTCCGTTCATGATGGAC
CGTGTCCTGTGGGAAAAAACCGGTCACTGGGACAACTACAAAGATGCAATGTTCACCAC
ATCTTCTGAGAACCGTGAATACTGCATTAAGCCGATGAACTGCCCGGGTCACGTACAAATT
TTCAACCAGGGGCTGAAGTCTTATCGCGATCTGCCGCTGCGTATGGCCGAGTTTGGTAG
CTGCCACCGTAACGAGCCGTCAGGTTCGCTGCATGGCCTGATGCGCGTGCGTGGATTTA
CCCAGGATGACGCGCATATCTTCTGTACTGAAGAACAAATTCGCGATGAAGTTAACGGAT
GTATCCGTTTAGTCTATGATATGTACAGCACTTTTGGCTTCGAGAAGATCGTCGTCAAACT
CTCCACTCGTCCTGAAAAACGTATTGGCAGCGACGAAATGTGGGATCGTGCTGAGGCGG
ACCTGGCGGTTGCGCTGGAAGAAAACAACATCCCGTTTGAATATCAACTGGGTGAAGGC
GCTTTCTACGGTCCGAAAATTGAATTTACCCTGTATGACTGCCTCGATCGTGCATGGCAGT
GCGGTACAGTACAGCTGGACTTCTCTTTGCCGTCTCGTCTGAGCGCTTCTTATGTAGGCG
AAGACAATGAACGTAAAGTACCGGTAATGATTCACCGCGCAATTCTGGGGTCGATGGAAC
GTTTCATCGGTATCCTGACCGAAGAGTTCGCTGGTTTCTTCCCGACCTGGCTTGCGCCG
GTTCAGGTTGTTATCATGAATATTACCGATTCACAGTCTGAATACGTTAACGAATTGACGCA
AAAACTATCAAATGCGGGCATTCGTGTTAAAGCAGACTTGAGAAATGAGAAGATTGGCTTT
AAAATCCGCGAGCACACTTTGCGTCGCGTCCCATATATGCTGGTCTGTGGTGATAAAGAG
GTGGAATCAGGCAAAGTTGCCGTTCGCACCCGCCGTGGTAAAGACCTGGGAAGCATGG
ACGTAAATGAAGTGATCGAGAAGCTGCAACAAGAGATTCGCAGCCGCAGTCTTAAACAAT
TGGAGGAACTCGAGCACCACCACCACCACCACTGA

The sequence of the *Ec*ThrRS_WT (242-642) protein:

MRDHRKIGKQLDLYHMQEEAPGMVFWHNDGWTIFRELEVFVRSKLKEYQYQEVKGPFMMD
RVLWEKTGHWDNYKDAMFTTSSENREYCIKPMNCPGHVQIFNQGLKSYRDLPLRMAEFGS
CHRNEPSGSLHGLMRVRGFTQDDAHIFCTEEQIRDEVNGCIRLVYDMYSTFGFEKIVVKLST
RPEKRIGSDEMWDRAEADLAVALEENNIPFEYQLGEGAFYGPKIEFTLYDCLDRAWQCGTVQ
LDFSLPSRLSASYVGEDNERKVPVMIHRAILGSMERFIGILTEEFAGFFPTWLAPVQVVIMNIT

DSQSEYVNELTQKLSNAGIRVKADLRNEKIGFKIREHTLRRVPYMLVCGDKEVESGKVAVRTR
RGKDLGSMDVNEVIEKLQQEIRSRSLKQLEELEHHHHHH*

The sequence of the open reading frame of the pObaO plasmid encoding ObaO_241-637_WT-6×His:

ATGCGTGACCACCGTAAACTGGCTAAACAGTTCGACCTGTTCCACCAGCAAGAAGAAG
CTCCAGGTATGGTCTTCTGGCATCCGAAAGGTTGGAGCCTGTGGCAGACCGTTGAACAG
TACATGCGTCGTGTTTATCGTGATGGCGGTTACCGTGAAGTTAAATCTCCGCAGGTACTG
GATTCTACTCTGTGGAAGAAGAGCGGCCACTGGGATAACTACAAAGAGAACATGTTCGTT
ACCGAATCCGAGAACCGTCAGTACGCACTGAAACCGATGAACTGTCCGGGTCACATCCA
AATCTTCAAACACGGTCTGCGTAGCCATCGTGAACTGCCGATCCGTTACGGTGAATTTGG
TGGCTGCCACCGTAACGAACCATCTGGCGCTCTGCACGGCATCATGCGTGTTCGTGCAT
TCACTCAAGATGATGGCCACATCTTCTGCACCGAAGAACAGATCGCGGCGGAAATCAAAG
CATTCCACTATCAGGCGGTTAAAGTTTACGCGGATTTCGGTTTCACCGACATCGCTGTTAA
GATCGCTCTGCGTCCGGAACCGGGTAAACGTCTGGGTTCCGACGAAGTTTGGGACAAAG
CGGAGAACCTGCTGCGTGAAGCGCTGTCTGAATGCGACGTTGAATGGGAAGAACTGCCA
GGCGAAGGTGCATTCTACAGTCCGAAGATCGAATACCACCTGCGTGATGCTATCGGTCGT
GAATGGCAGGTTGGTACTATGCAGGTTGACTACCACATGCCAGATCGTCTGGGTGCAGAA
TACGTTGATGAACACAGCCAGCGTCGTAAACCGGTTATGCTGCATCGTGCGATCGTGGGT
AGCCTGGAACGCTTTCTGGGTATCTTGATCGAACACCACGCAGGTCAGTTCCCGCTGTG
GCTGGCGCCGGTGCAGGCTATCGTGGTTACCGTTACCGACGCTCAGAACGATTACGCTG
ACCAGACTCGTAACGATCTGGTTCAGTTGGGCTTCCGTGTGGAAGCGGACCTGCGTAAC
GAGAAGATCGGCTACAAGATCCGTGAATCTACCTTGCAGCGTGTACCGTACCTGCTGGTA
GTTGGTGAACGTGAGAAAGAGAACGGCACCGTAACCGTTCGTTCTCGTGCGGGTGAAG
ACCTGGGTTCTATGACGATGGAAGCGCTGCATGCGTTTCTGCTGAACGAACAGTCTGCG
GGTGGTCTCGAGCACCACCACCACCACCACTGA

The sequence of the ObaO(241-637) protein:

MRDHRKLAKQFDLFHQQEEAPGMVFWHPKGWSLWQTVEQYMRRVYRDGGYREVKSPQ

VLDSTLWKKSGHWDNYKENMFVTESENRQYALKPMNCPGHIQIFKHGLRSHRELPIRYGEF
GGCHRNEPSGALHGIMRVRAFTQDDGHIFCTEEQIAAEIKAFHYQAVKVYADFGFTDIAVKIAL
RPEPGKRLGSDEVWDKAENLLREALSECDVEWEELPGEGAFYSPKIEYHLRDAIGREWQVG
TMQVDYHMPDRLGAEYVDEHSQRRKPVMLHRAIVGSLERFLGILIEHHAGQFPLWLAPVQAI
VVTVTDAQNDYADQTRNDLVQLGFRVEADLRNEKIGYKIRESTLQRVPYLLVVGEREKENGT
VTVRSRAGEDLGSMTMEALHAFLLNEQSAGGLEHHHHHH

Abbreviations

ThrRS	threonyl-tRNA synthetase
ObaO	a threonyl-tRNA synthetase homolog of *Pseudomonas fluorescens* which confers resistance to Obafluorin
WT	wild type
OB	obafluorin
BN	borrelidin
L-Thr	L-threonine
ATP	adenosine 5'-triphosphate
Tris	Tris(hydroxymethyl)aminomethane
MES	2-morpholinoethanesulphonic acid
HEPES	4-(2-Hydroxyethyl)-1-piperazine ethanesulfonic acid
MOPS	3-(*N*-Morpholino)propanesulfonic acid
MPD	2-methyl-2,4-pentanediol
PEG	polyethylene glycol
MME	monomethyl ester
TSA	thermal shift assay
Tm	mid-melting point
MD	molecular dynamics
DCCM	dynamic cross-correlation maps
$D_{R\text{-}A}$	distance between Arg363 and Ala460 of *E. coli* ThrRS
FEL	free energy landscape
Rg	radius of gyration
IC_{50}	half-maximal inhibitory concentration
SBVS	structure-based virtual screen
BTK	Bruton's Tyrosine Kinase
NRPS	non-ribosomal peptide synthetase
MoA	mechanism of action
PDB	protein data bank

V. Supplementary References

1. Consortium, T. U. UniProt: a worldwide hub of protein knowledge. *Nucleic Acids Research* **47**, D506-D515 (2018).
2. Bairoch, A. The ENZYME database in 2000. Nucleic Acids Research 28, 304-305 (2000).
3. Kumar, S., Stecher, G. & Tamura, K. MEGA7: Molecular Evolutionary Genetics Analysis Version 7.0 for Bigger Datasets. *Molecular Biology and Evolution* **33**, 1870-1874 (2016).
4. Letunic, I. & Bork, P. Interactive Tree of Life (iTOL) v6: recent updates to the phylogenetic tree display and annotation tool. *Nucleic Acids Research.* gkae268 (2024).
5. Sievers, F. & Higgins, D. G. Clustal Omega for making accurate alignments of many protein sequences. *Protein Science* **27**, 135-145 (2018).
6. Robert, X. & Gouet, P. Deciphering key features in protein structures with the new ENDscript server. *Nucleic Acids Research* **42**, W320-W324 (2014).
